# Membranous Dysmenorrhœa

**Published:** 1882-04

**Authors:** I. Stroinski

**Affiliations:** Chicago


### ARTICLE II.

MEMBRANOUS DYSMENORRHŒA. By I. STROINSKI, M.D. Chicago.

It is a rather difficult task to throw light upon this hitherto obscure disease, and it is my endeavor to write only after careful investigation of the sympathetic nerves of the abdominal system, a problem not very easily solved. My clinical observations of this disease have been extended. During a course of travel on both sides of the ocean, I have had the opportunity of making more examinations than many other physicians.

Virchow first endeavored to explain membranous dysmenorrhœa, and he called the product of the womb, the expelled membrane, "decidua menstruations;" a name maintained up to this day. He considered the process as a desquamative one, similar to that of the skin, and originating from a proliferation of the cellular tissue. Hausmann * declared the disease to be a "retrogradation of the germinal layer," in the sense of Darwin, and he thought the development of the membrane to be equivalent to that in pregnancy. Clarke and Williams opened a wider field; they proved the protuberances on the outer layer of the

* I would call the attention of R. Barnes, in London, and other authors, to the views of Hausmann, changed already in 1871, and given in the *Zeitschr f. Geburtsh* (Band I, Heft II.)

membrane to be elongations of the utricular glands, and taught that the membrane was in the same state of development as in pregnancy. Engelman and Kundrat enlarged upon these views by proving that the process was that of fatty degeneration, but they were mistaken in assuming the membrane to be in the same state as in pregnancy. Wyder has undoubtedly proved that the cells of the cellular tissue are in menstruation and membranous dysmenorrhœa different from those in pregnancy.

I had the opportunity of making experiments on the sheep on a large scale some years ago. Here the left spermatic ganglion is easily exposed while situated on the outer margin of the peritoneal duplicature, and it is easily separated by paring it from the adjacent spermatic vein and artery. Pressure (with a small clamp) on the ganglion excited the ovary, and the uterine membrane discharged a considerable quantity of a clear mucus. By continued pressure the blood-vessels of the ovary enlarge, and the ripening follicles swell, the uterine vessels enlarging but to a small extent, and the glands pouring out a considerable amount of mucus. A mild galvanic current, the positive pole inserted into the ganglion, the negative pole either into the trunk of the plexus or into the uterine membrane, excites the membrane to that state called irritation, and described below. A strong current causes all the incidents described below as continued irritation. Resection of the ganglion and a part of the spermatic plexus bring forth the most copious discharge on the opposite side of the uterine membrane, which is only surpassed by the discharge of fluid into the intestines after the resection of the mesenteric plexus.

Free phosphorus, applied either in the form of a paste or as a vapor, to the exposed ganglion, acts as a powerful agent on the nerves and the uterine membrane. After the application of the phosphorus to the ganglion, the adjacent parts being protected by waxed paper or cloth, the nerves will be found to be changed so that the axis cylinders look like hard cooked albumen, the uterine membrane being in the same state of fatty degeneration as in membranous dysmenorrhœa. The effect on the uterine membrane can be seen after ten minutes' action of the phosphorus vapor, but the operator has to be very careful not to come in contact with the vapors from the iron bottle. The fatty degeneration evoked by the phosphorus vapor will be partially restored to the normal state by applying a mild galvanic current to the ganglion and the uterine membrane. By applying the vapor to the uterine membrane, there will be seen a degeneration of all the sympathetic nerves supplying the uterus, and the degeneration in the spermatic plexus will reach the point of connection with the renal nerves. The application of phosphorus has been, in my hands, the only means of detecting a degeneration of the sympathetic nerves visible under the microscope, and I must assume that phosphorus-poisoning by the stomach acts directly upon the gastric sympathetic fibers, which by reflex action causes those lesions in the body found in phosphorus-poisoning; the blood dissolving the phosphorus to so small an extent as to be uninjured. The casting off of the uterine membrane in cholera will appear not so marvelous when it is remembered that a sudden reduction of the temperature in the lower parts of the body affects the intestines so as to cause diarrhœa.

In irritation, from whatever cause, the uterine membrane undergoes the same anatomical changes as does every other nutritive membrane. The epithelial cells of the utricular glands, which are of a columnar ciliated form, and which enclose a large nucleus of lucid appearance, containing prepared mucus, discharge the mucus, and the nucleus is restored by immigration of coarse granulated protoplasm. In continued irritation, the supply of protoplasm is not sufficient, and other substances, such as fatty matter, enter the cell, but the latter not being able to assimilate (digest) these substances, they are thrown out, and there is but an empty shell ; the cell is dead, and will be thrown off. The epithelial layer of the utricular glands is lined by a basement membrane of a protoplasmic character, supplying the epithelial cells. It disappears (is devoured) by continued irritation. The epithelial tissue of the membrane itself plays a rôle similar to that of the glands. The cellular tissue of the utricular glands consists of small round cells, filled with a protoplasmic nucleus, the latter containing mucus in a state of rest. These cells discharge the mucus in a state of irritation, and they fill with protoplasm ; but in continued irritation the protoplasm contracts, and the empty space is filled with other material, mostly of a fatty kind. The intercellular tissue of the glands takes on an inflated appearance, and causes the enlargement of the glands. But there are some small ovoid cells interspersed between the round cells, and they take no part whatever in irritation, but in gestation they emigrate, multiplying in a marvelous manner, take on a rounded shape, and form the greatest part of the decidua serotina.

The cellular tissue of the membrane itself undergoes a change similar to that of the utricular glands, but there is no prepared mucus contained in the cells. In irritation this tissue takes a negative part, but by continued irritation the protoplasm is partially discharged, and the cells fill with a turbid matter and oily drops; the intercellular tissue being swollen to a considerable degree. The highest point of fatty degeneration, that of induration, will be reached by phosphorus-poisoning and in membranous dysmenorrhœa. Here the epithelial tissue is changed to a lamellous, leather-like structure; the intercellular tissue to a true fibrous substance; the cells of the cellular tissue, in the glands as well as in the membrane itself, are filled with oily matter, or their contents thrown out entirely; the glands are very friable, and only the lower part of the glands, imbedded in the uterine substance, is in a healthy state. The blood-vessels of the upper layer are contracted so as to contain no blood; the membrane in its upper layer is dead.

From all my investigations, the full details of which cannot be described in these limits, I have formed the following theory of menstruation in general, and of membranous dysmenorrhæa in particular:

I consider it to be a law of nature that in every living individual, in plants as well as in animals, there are developed at certain times and at certain places one or more distinctly formed cells, which, mostly after impregnation by another but differently constructed cell of the same or another individual of the same species, develop an individual similar to that from which it is the offspring. The development (ripening) of the cell (ovum) before impregnation, I regard as a factor *per se*. This development (ripening) of the cell (ovum) reproduces in the affected individual a series of varying changes in the adjacent parts

(genital apparatus). These changes are always dependent upon the presence of a ripening ovum. In all the mammalia, including the human female, these cells (ova) are implanted from the epithelial germ layer, and each of these very numerous epithelial cells is capable of becoming a new individual. But nature dispenses her gifts in abundance, and thus but a small quantity of the ova ripen, and but a very few come in contact with the elected spermatozoön to form the zoösperm, *i. e.*, the new individual. The ripening ovum causes a proliferation of the epithelial tissue in the Graaffian follicle, with serous exudation into the same. The Graaffian follicle enlarges, and by its growth the ovarian nerves will be irritated. The growth continuing, the uterine fibers will be irritated by reflex action, and there will be a discharge from the uterine glands; the ovum prepares thus its food, the uterine milk, which is necessary for the developing impregnated ovum. In all the mammalia this process is always the same. But in the woman, endowed with a very sensitive system, nature makes an occasional *faux pas.* The continued irritation of the lower sympathetic system excites the upper part, and finally an over-stimulation of the vaso-motor center. The uterine blood-vessels become enlarged by the paresis of the vaso-motor center; they form ecchymoses and burst. Thus the original process is rendered indiscernible. Sometimes the sympathetic system at large is in a hyper-sensitive state, so as to excite the capillaries of other membranes (skin, bronchial membrane, conjunctiva, etc.), which will burst and discharge the blood from these places, in which a good Catholic may discern his "saints." The uterine membrane is decidedly of a nutritive character, the glands supplying the uterine milk for the impregnated ovum, the winding course of the blood-vessels being instituted for nourishing that part of the body which becomes a new individual (embryo).

The menstrual process in all the mammalia and the beginning of menstruation in women bear a striking resemblance to digestion, the minute anatomical changes in the uterine membrane being the same as in the gastric membrane, and I regard menstruation at large as a nutritive irritation excited by the reflex action from the irritated ovarian nerves. But in women nature makes a second *faux pas.* The uterine nerves being over-irritated and the glands being exhausted, there appears a fatty degeneration in the uterine membrane, so as to cast off a part or the whole membrane. But here we must find the limits between the physiological and the pathological state; between health and disease, by the clinical history.

It has been my lot to register in the last twelve years, while practicing in both hemispheres, thirty-five cases of membranous dysmenorrhœa, a number which seems to be incredible compared with the reports of others. But I must add immediately, that in fourteen of these cases the membrane has been cast off but once, with all the symptoms of an acute attack, the most cases occurring in the country. That the cast-off membrane was not an abortive decidua the following phenomena would teach me, but the microscope has always been my resource in detecting the characteristic form of the cells.

In a mill situated in a low valley, bordered by high mountains, where in every spring the valley was overflowed for weeks, I have seen three servant girls suffer from the disease, the lady of the house also suffering from it for fifteen years. I will relate one of these cases, the first to occur in my practice.

This servant had been delivered six months previously of a still-born child, the menses reappearing six weeks after the confinement. In scrubbing the front steps of the house in winter time, with bare feet and unprotected limbs, as is the general custom among the laboring women in Germany, she took cold, and had a slight fever in the night, with a chilly sensation creeping from her feet up to the abdomen, but the next morning she was quite well. In the next menstruation, there came on intense pains in the suprapubic region, which lasted for two days, followed by the expulsion of a membrane, and then a considerable flow of blood set in. The membrane was instantly recognized by the lady of the house, and I examined the girl and the membrane. There was no inflammation either in the meso or endometrium, and the uterus had the normal size. The ovaries were normally situated, and without pains. The membrane showed the well-known bag with the serrated opening on the os internum and the orifices of the Fallopian tubes. The openings of the glands were visible with the naked eye, and they had the mosaic-like appearance resembling the eye of an insect. Not being acquainted with the treatment of the disease at this time, I dismissed the girl. Two years later I saw her again, with a baby on the arm. She had had the same phenomena as long as she served in the mill (for six months), but by removing to a village in the mountains, the membrane slowly disappeared, and her lover having come home from the war, she married and became pregnant.

A very different course was that of another case. The only daughter of a wealthy farmer in Iowa went to a party, where she danced all night. In going home (about one mile) the girl took off her stockings and shoes, her feet being sore from the dance, and walked home barefoot. When in bed she felt a cold sensation extending from her feet to her bowels, and a fever set in, which lasted but a few hours. She rested the next day, and then went to work as usual. At the time of the next menstruation she awoke with terrible pains in the suprapubic region and in the bowels, which lasted for three days. The mother of the girl feared that something was the matter, but I could assure her that the girl was a virgin by removing the membrane from its place under the well-developed hymen by a pincette. The same symptoms reappeared with the next menstruation, and after four months the formerly healthy and well developed girl began to fail. There appeared red spots * on the chest and back, which were sometimes of the same intense color as in scarlatina. Contractions of whole groups of muscles set in, and the girl was hysterical and anæmic. A tour to the South relieved her somewhat, but after staying home for a short time the same phenomena reappeared. The hitherto refused local treatment was now accepted; one electrode inserted into the cavity of the uterus—the thick hymen being excised—the other alternately on the right and left ovarian region. A mild galvanic current was applied every second day. The next menstruation appeared without intense pain, and there were thrown off only small pieces of the membrane. The treatment was continued for four months, and the girl restored to her full health. She has married since, and borne children, without any relapse of the disease.

---

* Cohnheim, at Berlin, described these spots in a recently published paper, and he thought, from the same spots appearing in hysterical persons, the disease to be an offspring of hysteria.

A very remarkable course was taken by another case in one of our suburbs. An Irish woman suffered from membranous dysmenorrhœa for eight months. Alternately with the expulsion of the uterine membrane there were separated from the bowels, mostly from the colon, large pieces of mucous membrane under the same intense pains. After I had treated her but a few times, she took sick with a true erysipelas faciei (erysipelatous dermatitis), which assumed a severe grade, but she recovered. Since that time there has never been cast off a uterine membrane, and her menstruation is as regular as before.

It is very seldom that physiological experiments reveal the cause of the disease and indicate the treatment at the same time, but, as I have shown above, there will be a partial restoration of the uterine membrane after degeneration by the phosphorus vapor in applying a mild electric current to the spermatic plexus and the uterine membrane. I have treated a case of membranous dysmenorrhœa of sixteen years' standing by the galvanic current with the best success, a complete cure; but the treatment had to be continued for eighteen months before the uterine nerves were fully restored.

Cases of a shorter duration demanded, of course, a shorter treatment; and I can say that I have succeeded in every case where the treatment had been kept up for the time necessary to complete a cure.

The inflammatory symptoms, as meso, or parametritis, chronic ovaritis, salpingitis, etc., I have found to be connected with the disease when the membrane had been expelled for a certain length of time, the nerves of the ovary, and those supplying the parenchyma of the uterus, or the ligaments and Fallopian tubes, becoming affected more or less. But in recent cases—and I have examined nineteen such—the uterus and the ovaries were painless, *i. e.*, without inflammation. I must therefore declare the disease *not* to be of an inflammatory character, as the most authors describe it. All the symptoms which will be found in old cases are the *result* of the disease, but not its cause.

As to the name of the disease, I will call it what it is—a paresis of the uterine nerves, and especially those springing from the spermatic plexus. I will leave it to the gynæcologists of Athens to endow it with a proper Greek title.

